# Supplementary material for: The initial charge separation step in oxygenic photosynthesis
Source: Nat Commun. 2022 Apr 27;13:2275. doi: 10.1038/s41467-022-29983-1 (PMC9046298; doi:10.1038/s41467-022-29983-1)
Supplement: Supplementary file 1 — Supplementary Information [file 41467_2022_29983_MOESM1_ESM.pdf]

Supplementary Information

The initial charge separation step in oxygenic photosynthesis

Yusuke Yoneda,[1,2,5] Eric A. Arsenault,[1,2,3] Shiun-Jr Yang,[1,2] Kaydren Orcutt,[1,2] Masakazu Iwai[2,4] and Graham R. Fleming[1,2,3]*

[1]*Department of Chemistry, University of California, Berkeley, CA 94720, USA*

[2]*Molecular Biophysics and Integrated Bioimaging Division, Lawrence Berkeley National Laboratory, Berkeley, CA 94720, USA*

[3]*Kavli Energy Nanoscience Institute at Berkeley, Berkeley, CA 94720, USA*

[4]*Department of Plant and Microbial Biology, University of California, Berkeley, CA 94720, USA*

[5]*Present Address: Research Center of Integrative Molecular Systems, Institute for Molecular Science, National Institute of Natural Sciences, Okazaki, Aichi, 444-8585, Japan*

[*]grfleming@lbl.gov

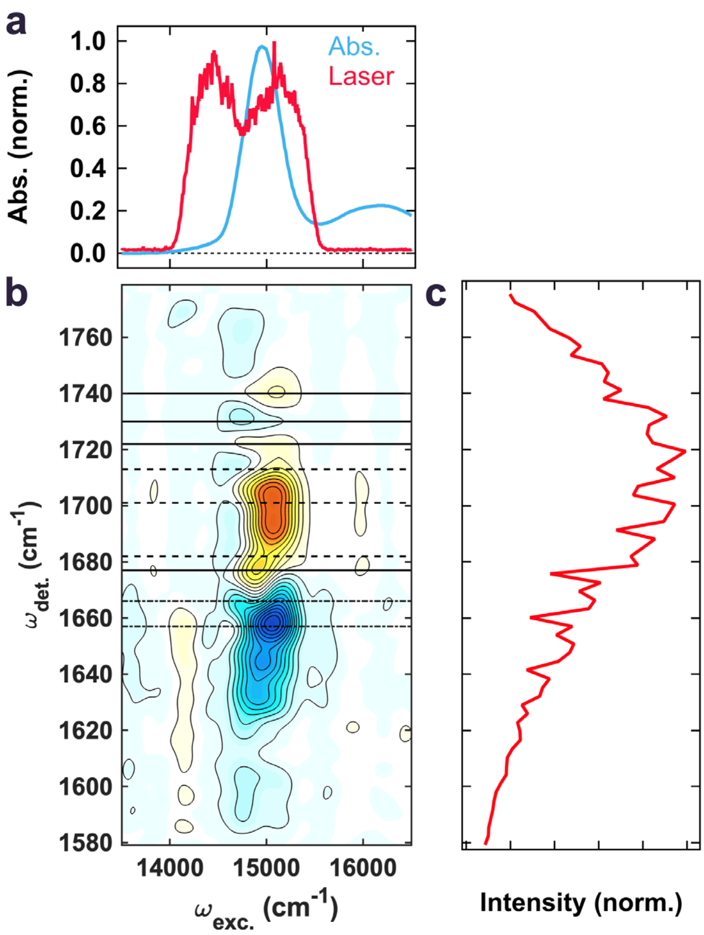

Supplementary Figure 1. Linear absorption spectrum of the PSII-RC (blue) and the spectrum of visible excitation laser pulse (red) (a). 2DEV spectrum of the PSII-RC at T = 180 fs (b). The horizontal solid, dotted, and dash-dotted lines follow the same convention as in Figure 2 in the main manuscript. The spectrum of the IR probe laser pulse is shown in (c).

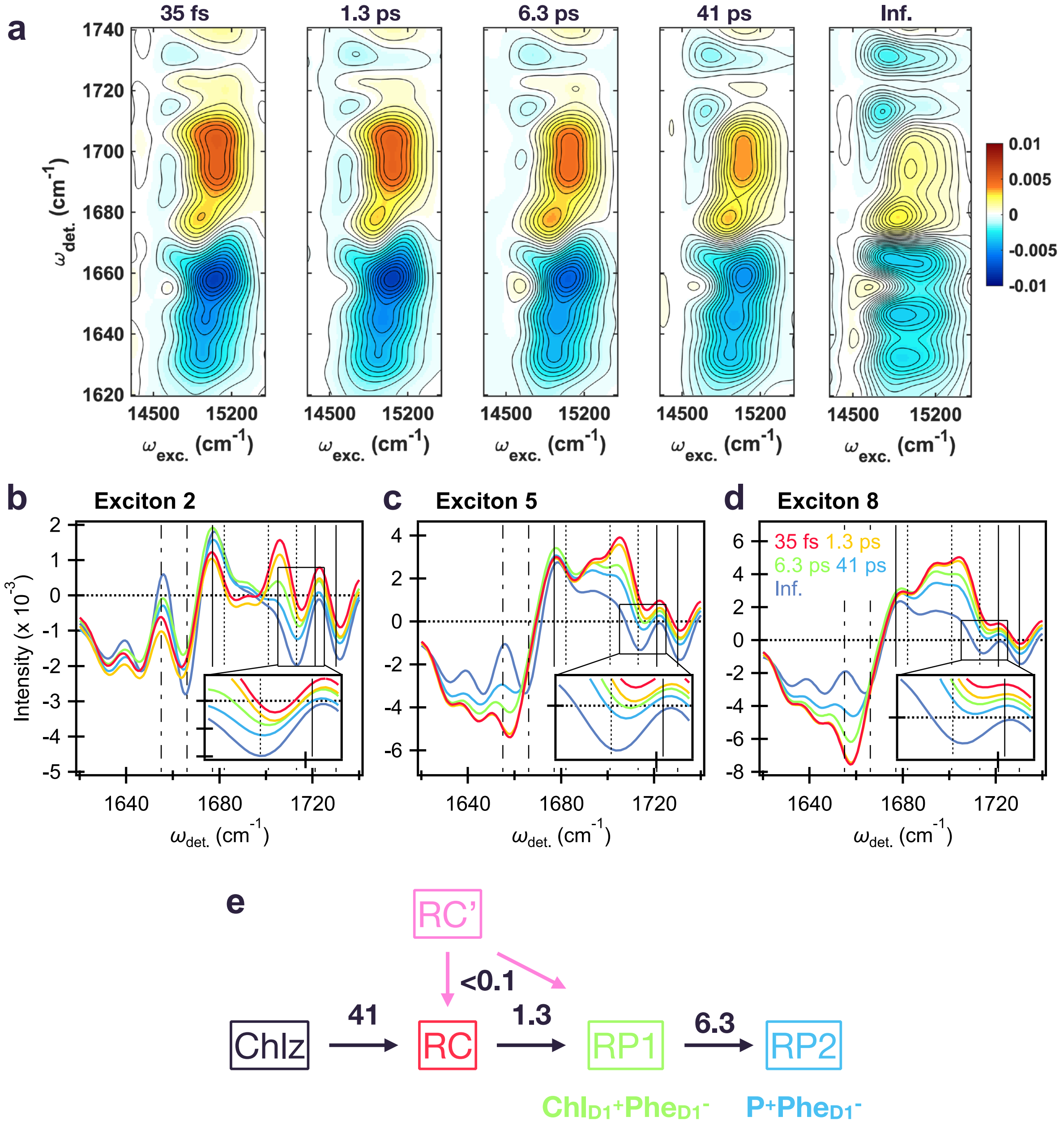

Supplementary Figure 2. Two dimensional-evolution associated difference spectra (2D-EADS) of the PSII-RC (a). Five components were required for a reasonable fit with time constants of 35 fs, 1.3 ps, 6.3 ps, 41 ps, and a non-decaying offset component (longer than current detection time range of 100 ps). Each 2D-EADS evolves into the next one with the time constants listed above. Contour levels are drawn in 5% intervals. The time-dependent evolution

of excitons 2, 5, and 8 are shown in (b)-(d), respectively. The vertical solid, dotted, and dash-dotted lines follow the same convention as in Figure 2 in the main manuscript. A simplified scheme for the excited state dynamics of the PSII-RC based on the result of 2D-EADS analysis is shown in (e). It should be noted that this scheme is not intended to imply that the dynamics of the PSII-RC follow a series of sequential, irreversible steps, but rather to grossly summarize the results of the 2D-EADS.
